# Identification of Snails and *Schistosoma* of Medical Importance *via* Convolutional Neural Networks: A Proof-of-Concept Application for Human Schistosomiasis

**DOI:** 10.3389/fpubh.2021.642895

**Published:** 2021-07-15

**Authors:** Krti Tallam, Zac Yung-Chun Liu, Andrew J. Chamberlin, Isabel J. Jones, Pretom Shome, Gilles Riveau, Raphael A. Ndione, Lydie Bandagny, Nicolas Jouanard, Paul Van Eck, Ton Ngo, Susanne H. Sokolow, Giulio A. De Leo

**Affiliations:** ^1^Hopkins Marine Station, Stanford University, Pacific Grove, CA, United States; ^2^Centre de Recherche Biomédicale Espoir pour la Santé, Saint-Louis, Senegal; ^3^Univ Lille, Centre National de la Recherche Scientifique (CNRS), Institut National de la Santé et de la Recherche Médicale (INSERM), Centre Hospitalier Universitaire (CHU) Lille, Institut Pasteur de Lille, U1019-Unité Mixte de Recherche (UMR) 9017-CIIL-Center for Infection and Immunity of Lille, Lille, France; ^4^Station d'Innovation Aquacole (SIA), à Université Gaston Berger, Saint-Louis, Senegal; ^5^International Business Machines Corporation (IBM) Silicon Valley Lab, San Jose, CA, United States; ^6^Department of Ecology Evolution and Marine Biology, University of California, Santa Barbara, Santa Barbara, CA, United States; ^7^Woods Institute for the Environment, Stanford University, Pacific Grove, CA, United States

**Keywords:** computer vision & image processing, schistosomiais, neglected tropical disease, deep learning - artificial neural network, image classification

## Abstract

In recent decades, computer vision has proven remarkably effective in addressing diverse issues in public health, from determining the diagnosis, prognosis, and treatment of diseases in humans to predicting infectious disease outbreaks. Here, we investigate whether convolutional neural networks (CNNs) can also demonstrate effectiveness in classifying the environmental stages of parasites of public health importance and their invertebrate hosts. We used schistosomiasis as a reference model. Schistosomiasis is a debilitating parasitic disease transmitted to humans *via* snail intermediate hosts. The parasite affects more than 200 million people in tropical and subtropical regions. We trained our CNN, a feed-forward neural network, on a limited dataset of 5,500 images of snails and 5,100 images of cercariae obtained from schistosomiasis transmission sites in the Senegal River Basin, a region in western Africa that is hyper-endemic for the disease. The image set included both images of two snail genera that are relevant to schistosomiasis transmission – that is, *Bulinus* spp. and *Biomphalaria pfeifferi* – as well as snail images that are non-component hosts for human schistosomiasis. Cercariae shed from *Bi. pfeifferi and Bulinus* spp. snails were classified into 11 categories, of which only two, *S. haematobium* and *S. mansoni*, are major etiological agents of human schistosomiasis. The algorithms, trained on 80% of the snail and parasite dataset, achieved 99% and 91% accuracy for snail and parasite classification, respectively, when used on the hold-out validation dataset – a performance comparable to that of experienced parasitologists. The promising results of this proof-of-concept study suggests that this CNN model, and potentially similar replicable models, have the potential to support the classification of snails and parasite of medical importance. In remote field settings where machine learning algorithms can be deployed on cost-effective and widely used mobile devices, such as smartphones, these models can be a valuable complement to laboratory identification by trained technicians. Future efforts must be dedicated to increasing dataset sizes for model training and validation, as well as testing these algorithms in diverse transmission settings and geographies.

## Introduction

**Figure 1:**
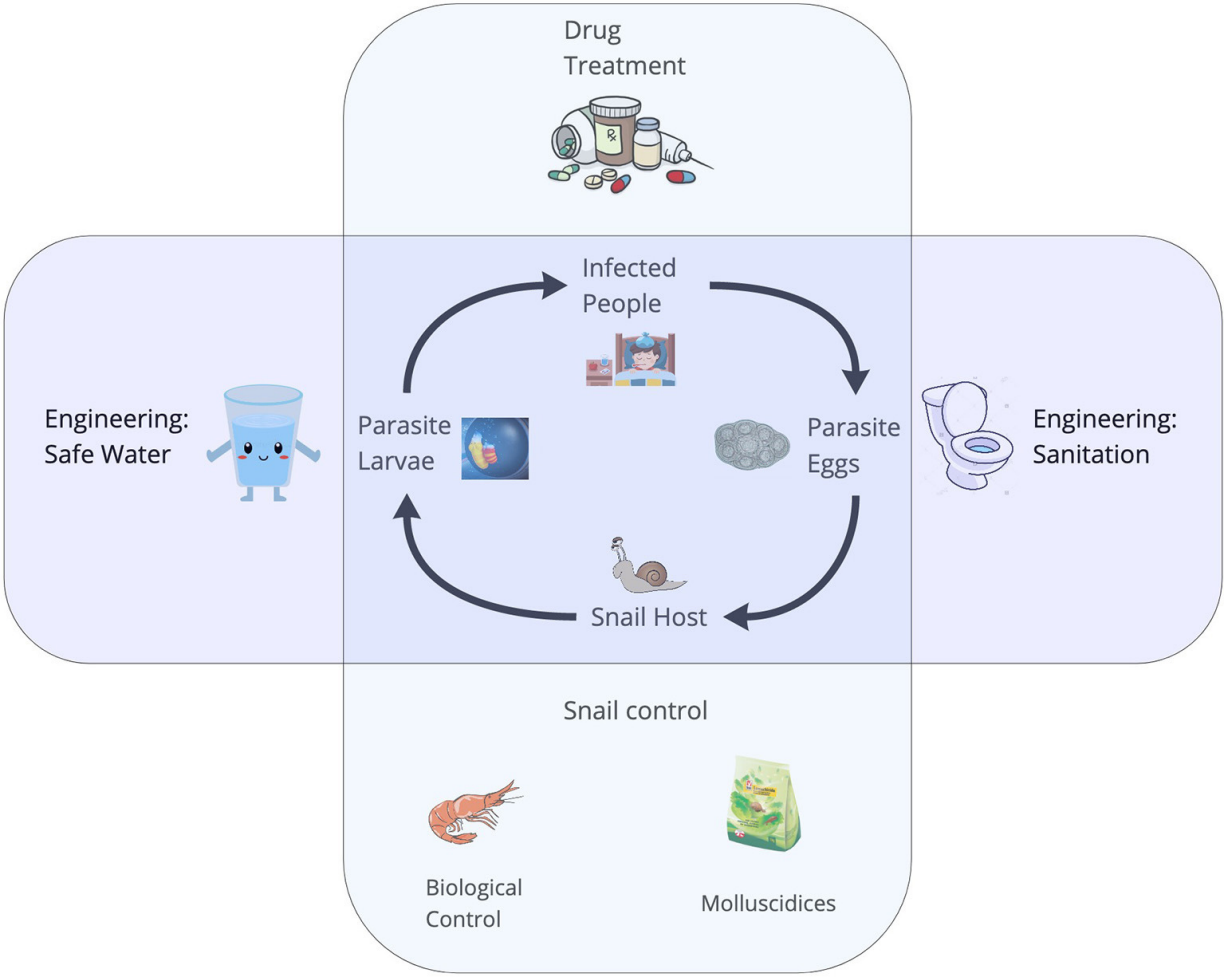
The life cycle of *Schistosoma* spp. The adult worms live and reproduce sexually within the human host and their eggs are released in the feces. In the environment, eggs must reach the water, and under appropriate conditions miracidia hatch and seek an intermediate freshwater snail host in the surroundings. The larval stages of the worms develop *via* asexual reproduction. Cercariae are the free-swimming larvae that are released from the snails and seek human hosts, completing the lifecycle.

Parasitic diseases of poverty, including schistosomiasis, onchocerciasis, lymphatic filariasis, and malaria, afflict billions of people worldwide (1). Many diseases of poverty have complex life cycles, whereby, trematodes require more than one host to complete their life cycles. Diseases of poverty include vectorborne diseases (i.e., malaria or arboviruses) and food-, soil-, or waterborne diseases involving intermediate hosts (e.g., schistosomiasis, food-borne trematodiasis). People living poverty settings often engage in physically-demanding work, such as subsistence agriculture or traveling long distances to fetch fresh water, exposing them to cercariae and pathogens embedded in the environment (2). Due to a lack of vaccines for many diseases of poverty, and inconsistent access to the few existing vaccines, the control of most diseases of poverty largely depends upon the ability to detect the distribution and abundance of cercariae of medical importance in the environment, as well as detection and mapping of the distribution, abundance, and infection status of their non-human hosts. Schistosomiasis, a parasitic disease of poverty afflicting more than 200 million people worldwide (1, 3) – with the vast majority in sub-Saharan Africa – is a disease of poverty with a complex life cycle (4) involving specific freshwater snail species as intermediate hosts (Figure 1). The two most important species causing human schistosomiasis in sub-Saharan Africa, *Schistosoma haematobium* and *S. mansoni*, are transmitted by snails belonging to two different genera of snails: *Bulinus* spp. and *Biomphalaria* spp., respectively. Several other snail species often co-occur in transmission sites where schistosomiasis is endemic. Snails that are intermediate hosts of schistosoma can be hosts for a wide range of trematodes, of which only few are of medical importance (5). In resource-limited settings, control strategies – which largely rely on mass drug administration – can be more effective when accompanied by environmental interventions, like targeted snail control (6). Therefore, reliable and rapid detection of schistosome cercariae and their intermediate host snails in water bodies is an urgent public health priority to identify where environmental interventions should be focused. This is especially crucial as environmental change – including climate change and the expansion of dams and irrigation schemes – is expected to alter the geographic distribution of schistosomes and their snail hosts (1).

Current protocols to sidentify snails and cercariae of medical importance requires molecular analysis of parasite or snail tissue (which is often inaccessible or prohibitively expensive in low-income settings), or visual identification of cercariae and snails by experienced parasitologists. This is a labor-intensive task that requires a great deal of expertise to tease apart multiple snail species that share the same freshwater habitat where schistosomiasis transmission occurs, and multiple parasite species that can infect the same snail hosts. Indeed, wide variety of livestock and wildlife cercariae, some of which are morphologically similar to (and in some cases, even indistinguishable from) human schistosome larvae (7), use schistosome intermediate host snail species to complete their cycles. Consequently, misidentification of snail infections as human schistosomes, when, in fact, they are cryptic species that do not harm people, may interfere with efficiently targeting environmental control activities to areas of high transmission.

Here we explore whether computer vision, specifically machine learning (ML) algorithms, can support the rapid identification of snails and cercariae of medical importance. ML is rapidly being developed for use in medicine and public health, where it has been already applied to tasks as diverse as early detection of brain tumors (5) and cancer and studies of health equity (8). Convolutional neural networks (CNN), a special class of ML algorithms, have proven to be particularly efficient in reading x-rays and identifying possible pathologies to a high level of accuracy (9). This progress has been possible because of the availability of large sets of digitized images labeled by experienced radiologists and physicians and often validated through clinical studies (10). Such resources are not yet available for the cercariae and hosts causing neglected tropical diseases, or schistosomiasis specifically: images cannot be scraped from the web and digital imagery repositories of snails and cercariae are generally not available. Even when they exist (11), they are usually limited to a few thousand images at most – just a fraction of the labeled x-rays available in the medical system.

The goal of this work is to assess how effectively CNNs can classify *Schistosoma* cercariae and their intermediate host snails when trained on a small and unbalanced imagery dataset. Specifically, we used 5,500 images of snails and 5,100 images of genetically identified cercariae from a sample of more 9,000 snails gathered in the lower basin of the Senegal River between 2015 and 2019 (11) to train a CNN algorithm and assess its accuracy. The set included images of host and non-host species for human schistosomiasis. More than 20 parasitic species were identified through shedding and dissection in the competent species of *Bulinus* and *Biomphalaria* snails from the study sites (11).

The paper is structured as follows: we first provide a detailed description of the training dataset, then present the CNN algorithm, and assess its accuracy when applied to the validation dataset. We then contrast this accuracy with the accuracy of a trained parasitologists' ability to classify the snails and cercariae. Additionally, we present an open-access web application where any individual can deploy the trained algorithm. Finally, we discuss future development and possible use-cases we anticipate as significant expansions of the training dataset in the future.

## Materials and Methods

### Datasets

During the environmental monitoring of snails and cercariae in the Senegal River Basin between 2015 to 2019, we collected 5,543 images of snails of medical importance categorized as follows – (1) *Bulinus globosus* and *Bulinus truncatus*, (2) *Biomphalaria pfeiffer*, (3) *Radix natalensis*, and (4) *Melanoides* spp. A total of 5,140 images of cercariae belonging to 11 morphotypes were obtained from the intermediate hosts of human schistosomiasis, *Biomphalaria pfeifferi* and *Bulinus* spp. These species were encountered and dissected. More specifically, there are 4 species of *Bulinus* (*Bu. globosus, Bu. truncatus, Bu. senegalensis* and *Bu. forskalii*), *Biomphalaria pfeiffer, Radix natalensis* (formerly known as *Lymnaea natalensis*) (12), and *Melanoides* spp. We had such rare occurrences of *Bu. senegalensis* and *Bu. forskalii* at these sites that they were not used in the training set. Nonetheless, *Bu. senegalensis* and *Bu. forskalii* are distinguishable by shell morphology down to species level.

**Figure 2:**
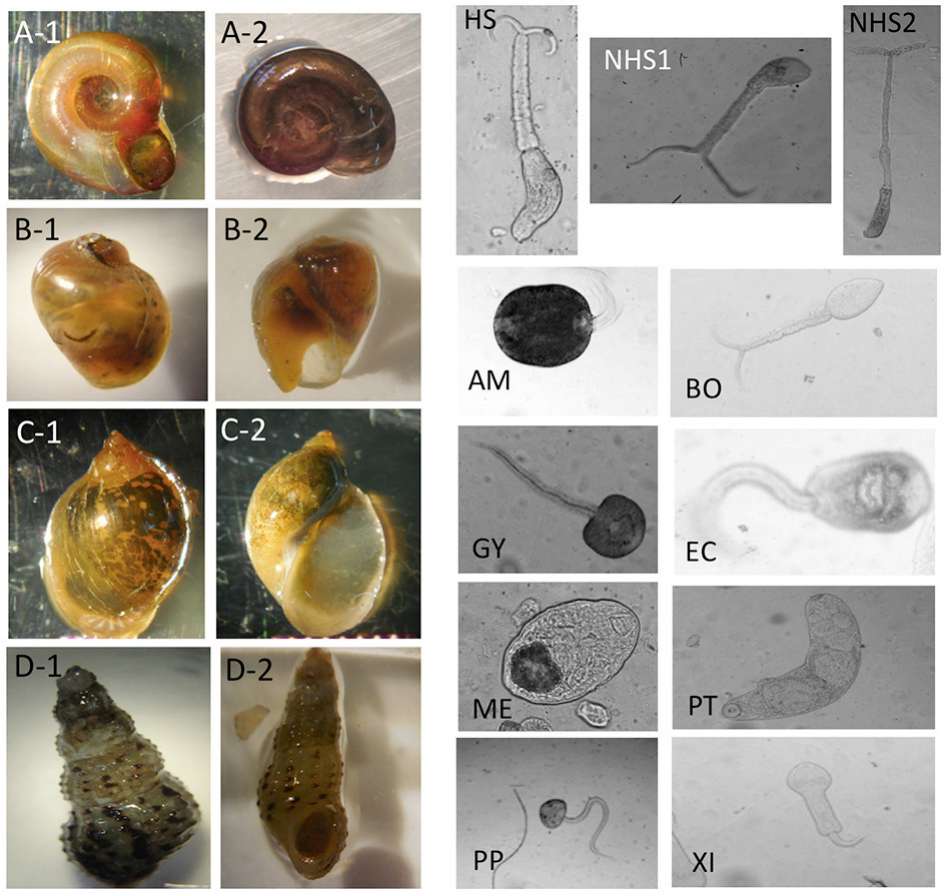
Image examples for snail and parasite categories. For snail categories, **(A-1,A-2)**: *Biomphalaria*. **(B-1,B-2)**: *Bulinus*. **(C-1,C-2)**: *Radix natalensis*. **(D-1,D-2)**: *Melanoides* spp. For parasite categories, HS, Human-schisto; NHS1, Nonhuman-schisto forktail type I; NHS2, Nonhuman-schisto forktail type II; AM, Amphistome cercariae; BO, *Schistosoma bovis*; EC, Echinostome cercariae; GY, Gymnocephalus cercariae; ME, Metacercaria; PP, Parapleurolophocercous cercariae; PT, Parthenitae; XI, Xiphidiocercariae.

Table 1 summarizes the numbers of images in each category and Figure 2 shows examples for each snail and parasite category in our dataset. Snail photographs were taken by mobile phone devices and cameras with similar resolution (of at least 1,024 × 768 pixels) and taken from similar angles, backgrounds, and lighting through the ocular lens of a dissecting microscope at 10–40× magnification. Parasite images were taken from the same variety of camera sensors through compound microscopes, and included images at 40× , 100× , and 400× magnification, with the same uniformity in background setting and ambient lighting. Some images were obtained from an intraocular camera, while some were from a digital single-lens reflex camera, a camera superior to cell phone cameras and more commonly used to image cercariae. The variety of high-quality cameras enabled the team to capture the necessary imagery, which worked well in training our model. In addition, all parasite images were processed to grayscale. The removal of color was performed to further decrease confounding results such as lighting and time-of-day artifacts that could affect computer vision results. The detailed protocols for image collection and quality control are provided in the supplemental materials (see Supplementary Appendix 1). Initial identification of snails and cercariae was performed by three trained parasitologists in the field. Our team only used images of cercariae that were genetically fingerprinted (which included only the *furcocercous cercariae*) or that were unequivocal in classification (11). If there was uncertainty among technicians in specifying a morphotype, genetic fingerprinting (11) results were obtained and analyzed. The protocol for image collection is provided in the supplemental materials (see Supplementary Appendix 1).

**Table 1:** Summary of numbers of images for each snail genus and parasite morphotype; numbers of images for the split of training/test (80%) and hold-out validation set (20%).

**Snail category**	***Biomphalaria***		***Bulinus***			***Radix natalensis***			***Melanoides*** **spp**.			**TOT**
Number of images	466		4,215			725			137			5,543
Training/test	374		3,372			580			109			4,435
Hold-out validation	92		843			145			28			1,108
**Parasite category**	**HS**	**NHS1**	**NHS2**	**AM**	**BO**	**EC**	**GY**	**ME**	**PP**	**PT**	**XI**	**TOT**
Number of images	1,008	638	107	442	224	332	152	196	806	231	1,004	5,140
Training/test	806	510	86	354	179	266	121	156	645	185	803	4,111
Hold-out validation	202	128	21	88	45	66	31	40	161	46	201	1,029

*Abbreviations of cercariae categorization as follow, HS, Human-schisto; NHS1, Nonhuman- schisto forktail type I; NHS2, Nonhuman- schisto forktail type II; AM, Amphistome cercariae; BO, Schistosoma bovis; EC, Echinostome cercariae; GY, Gymnocephalus cercariae; ME, Metacercaria; PP, Parapleurolophocercous cercariae; PT, Parthenitae; XI, Xiphidiocercariae*.

Accuracy of field identifications were verified by a molecular barcoding technique: at the time of shedding or dissection, all fork-tailed cercariae liberated from snails were placed individually on Whatman FTA© cards and sequenced to distinguish human-infective strains, including *Schistosoma haematobium* and *S. haematobium–bovis* hybrids, from cattle- or bird-infecting strains including *S. bovis* and other non-human furcocercous (fork-tailed) trematode species (13). The identification of the cercariae was based on multi-locus analyses with one mitochondrial (*cox*1) and two nuclear (*ITS*1 + 2 and 18S) genes, as described (11). Cercariae on FTA cards were accessioned into the Schistosomiasis Collection at the Natural History Museum (SCAN) (14).

All *Biomphaslaria* and *Bulinus* spp. snails were also identified to species by both trained parasitologists and genetic barcoding in a previous study (11). For the purposes of this work, *Bu. globosus* and *Bu. truncatus* were grouped in a single category, the *Bulinus* species complex that hosts human schistosomes in this region, and all the rest of the snails were visually classified to species using morphological keys. The total genomic DNA was isolated from a small amount of snail tissue using the DNeasy Blood and Tissue kit (Qiagen, UK) according to the manufacturer's instructions. Amplification of a partial cytochrome oxidase 1 (*cox1*) sequence was carried out on snail tissue stored in ethanol (15). PCR and sequencing conditions were chosen as previously published (16). Sequencing was performed on an Applied Biosystems 3730XL analyser (Life Technologies, UK) (11). The field ID guidelines for morphologies of snails and cercariae are provided in the supplemental materials (see Supplementary Appendix 2).

For imagery of snails and cercariae, we strategically avoided extensive processing of images, given that our goal was to create a CNN that was capable of classifying images of variable quality and resolution. We anticipate that for a future deployment, many technicians will be taking pictures directly from mobile devices in the field, hence, we decided to work with imagery that reflects true fieldwork conditions. Therefore, in the preparation of our training data, images that were severely blurry were removed from the test and validation sets but were still used in training. For each category of snails and cercariae, we divided the dataset into a training/test set and hold-out validation set, containing a split of 80 and 20% of the total images, respectively (Table 1). There was no overlap between the training set and the validation set. Both the training and the test sets were randomly divided into the standard 80–20% split in each training epoch and were used to perform 10-fold cross-validation on multiple train-test splits to ensure the consistency of model performance.

### Training Algorithm

Deep learning algorithms with recent advances in computation and large datasets have been shown to be comparable with, and even exceed, human performance in various object recognition and computer vision tasks, including applications to diagnose human disease [e.g., ImageNet challenge (17), breast cancer histology images (18), and skin cancer images (19). Convolutional neural networks (CNNs) learn key features directly from the training images by the optimization of the classification loss function (20, 21) and therefore have minimal need for *a priori* knowledge to design a classification system. Thus, the performance is less biased by the assumptions of the researchers (17, 21).

Since, our dataset was considered relatively small in the field of deep learning, we utilized transfer learning in this study (22). Transfer learning is the process of exporting knowledge from previously learned sources to a target task (23). In this study, we tested seven state-of-the-art pre-trained models in computer vision as a starting point and applied them to classify the images of freshwater snails and cercariae. The pre-trained models we tested for this study were (1) VGG16 and VGG19 (24), (2) Inception V3 (25) and Xception (26), (3) ResNet50 and ResNet101 (27) and (4) InceptionResNet V2 (28). These pre-trained models were all trained on approximately 1.28 million images with 1,000 categories, a computer vision benchmark dataset, called *ImageNet*.

VGG16 and VGG19 pre-trained models (24) are 16-layer and 19-layer weight CNNs using an architecture with very small (3 × 3) convolution filters. VGG16 and VGG19 were developed by the VGG team (Visual Geometry Group at the University of Oxford) in the ImageNet Large Scale Visual Recognition Challenge. Inception V3 (25) and Xception (26) pre-trained models were mainly developed by Google Inc. Inception V3 implements factorized convolutions and aggressive regularization, while Xception improves Inception modules by replacing it with depth-wise separable convolutions, which slightly outperforms Inception V3 on the ImageNet dataset. ResNet50 and ResNet101 (27) are pre-trained models with residual functions reformulated in the CNN layers, with 50 and 101 residual net layers, respectively. InceptionResNet V2 (28) is a hybrid CNN network, combining the Inception architecture with residual connections. The details of the pre-trained model architecture and design can be found in the individual cited publication.

We tested and trained seven selected computer vision algorithms, *via* transfer learning, with ImageNet pre-trained weights on our snail and parasite images (22, 23). The network architecture established here has convolutional-pooling layer pairs (max-pooling), followed by a fully connected network (29). The CNN is trained end-to-end directly from image labels and raw pixels, with a selected group of networks for photographic images of snails and another separated network for the microscopic images of cercariae.

We experimented several input image sizes and adopted the training patches as 128 × 128 pixels for input layers; the patch size was sufficient to cover the relevant structures and morphologies of snail and cercariae. In each experiment, we first initialized the weights with the pre-trained network on ImageNet dataset, then froze the bottom of the network, and proceeded to train the “top” of the selected convolutional networks. The top layer of the selected convolutional networks correlates to the “head” of the network. The weights of the top layer are most directly influenced by the labels. This is the layer that effectively produces the probabilities that the model is seeking to determine as output. The fully connected layers were composed of Rectified Linear Units (i.e., the ReLU activation function), to avoid vanishing gradients and to improve the training speed (30). The output layer was composed of four neurons for snail classification, corresponding to each of the four categories that are normalized with a softmax activation function. For the parasite classification task, 11 neurons were set up in the same manner. The model was trained with 80% of the training/test set, and validated on the 20% remaining images (i.e., hold-out validation set) that were not used for training (Table 1). It should be noted here that the test set is randomly selected for each epoch (that is, the measure of the number of times all of the training images are used once to update the network weights) (29). The network weights were initialized randomly, and an adaptive learning rate gradient-descent back-propagation algorithm (30) was used for weight update. Here we selected categorical cross-entropy as a loss function in the model. In these two classification tasks, the CNN outputs a probability distribution over four categories of snails and 11 categories of cercariae.

Given our comparably small dataset in light of more recent studies using CNNs for image recognition tasks (17), we applied the techniques of dropout, regularization, and data augmentation (details in the following section) to overcome the overfitting of training data (29). We implemented the CNN model with seven selected networks in a Python environment with the Keras application package (31), and Google's deep learning framework, TensorFlow (32).

### Data Augmentation

Data augmentation is an effective way to reduce overfitting. We applied a data augmentation approach using *rotation* and *shifting* (33) to generate more images for the snail and parasite datasets. Mirroring was not used for *Bulinus* and *R. natalensis* due to the diagnostic value of their coil orientation (i.e., *Bulinus* is sinistral and *Radix* is dextral). We also applied Gaussian noise to the background of the parasite training images (34) to ensure that the model did not learn background artifacts, but focused only on learning the morphologies of the parasite objects. In this study, we implemented a Keras-defined generator for automating data augmentation (31) on the fly; every item of every batch was randomly altered according to the following settings: (1) rotation range = 20, (2) width shift range = 0.2, (3) height shift range = 0.2, and (4) shear range = 0.15. In practice, rotations and shifting allowed us to increase the size of the dataset without deteriorating its quality. The data augmentation used here further improved the datasets and the CNN's prediction performance.

### Model Optimization

In our training process, we tested seven selected CNN models subsequently (refer to Training Algorithm section for pre-trained model types) and experimented a set of hyperparameters for the learning algorithms, which included image input size, training epoch, batch size, dropout rate, and learning rate. We utilized these parameters to obtain a final accuracy after every run. We conducted a grid search hyperparameter tuning to obtain the optimal set of parameters whose values are used to control the learning process. The specified subset of each hyperparameter is summarized in Table 2. We recorded all the experiments performed and reported the best performing model along with the optimal hyperparameter set in the Results section. As shown in the Supplementary Material, we tested parameters in the following order: epoch values, analyzing accuracy results with epochs between 5 and 150. We found that models that run on a higher number of epochs were producing consistent increases in accuracy percentages and that epoch accuracy stabilized at 150. We tested image input sizes between values of 64 and 256, finding that the accuracy of the model when these two parameters were manipulated, capped at around 89.70%. Note that between image input size values of 128–256, we noticed the overall accuracy percentages of the models remain nearly the same. Hence, we took 128 as our standard value for image input size (to carry forward further model-testing).

**Table 2:** Results of the identification of snails and cercaria by the best CNN model.

	**TP**	**TN**	**FP**	**FN**	**Sensitivity (%)**	**Specificity (%)**	**F1 score**
**Snail category**							
*Biomphalaria*	92	1,020	0	0	100.00	100.00	1.00
*Bulinus*	843	265	4	0	100.00	98.51	0.99
*Radix natalensis*	145	963	0	4	97.31	100.00	0.99
*Melanoides* spp.	28	1,084	0	0	100.00	100.00	1.00
**Parasite category**							
Schisto	170	801	14	30	85.00	98.28	88.54
Non-human forktail	173	807	19	16	91.53	97.69	90.81
Other trematodes	619	369	20	7	98.81	94.86	97.88

*TP, true positive; TN, true negative; FP, false positive; FN, false negative*.

Batch size accuracy stabilized at around 32, while dropout rate accuracy stabilized at around 0.45. Learning rate value tests ranged from 0.00001 to 0.1; results demonstrated the highest accuracy percentages at 0.001, with our model accuracy at up to a rough 90.50% with the current variable inputs. Some of the model parameters were co-dependent or dependent on one another; for instance, if the learning rate was too low, the number of epochs could affect the final accuracy percentages; so, all co-dependent variables and relationships were examined and tested for.

The last step was to take all the best models in each of the grouped subtests described above. We listed 20 out of the 161 model accuracy values (with each accuracy value being derived from the best of 5 runs) and then re-ran those top 20 models (5 times each) to produce the highest accuracy values of the model optimization process. We discovered a final best pre-trained model of InceptionResNetV2 with an image input size of 128, at 150 epochs, a batch size of 32, a dropout rate of 0.45, and a learning rate of 0.001, to produce a final accuracy of 91.21%. We tested and verified all models, and the weight and confusion matrix files are all in our Supplementary Material.

### Model Deployment

After building and training the CNN model, we then deployed the best performing model and network weights to establish a web application for inference using TensorFlow.js (35), a library used for executing ML algorithms in JavaScript. TensorFlow.js is compatible with the Python-based TensorFlow and Keras APIs, allowing our Keras model to be converted to a JavaScript format that can be run in a web browser. This makes our web application accessible on any device with a modern browser, including on both smartphones and common laptop computers. Using browser storage and caching APIs, the web application can even be used in areas with no internet connectivity.

### Ethics Statement

Freshwater snails were collected in collaboration with the Centre de Recherche Biomédicale Espoir pour la Santé in Senegal, who obtained the permission to conduct the field snail collection from The Direction de l'Environement et des Etablissements Classés with the identification number “N°002302 MEDD/DEEC/yn.” In this study, we used photos of snails for the machine learning model training and validation, and relied on those photos and snail and parasite molecular identities that were acquired in the course of a previous field study described in (11).

## Results

**Figure 3:**
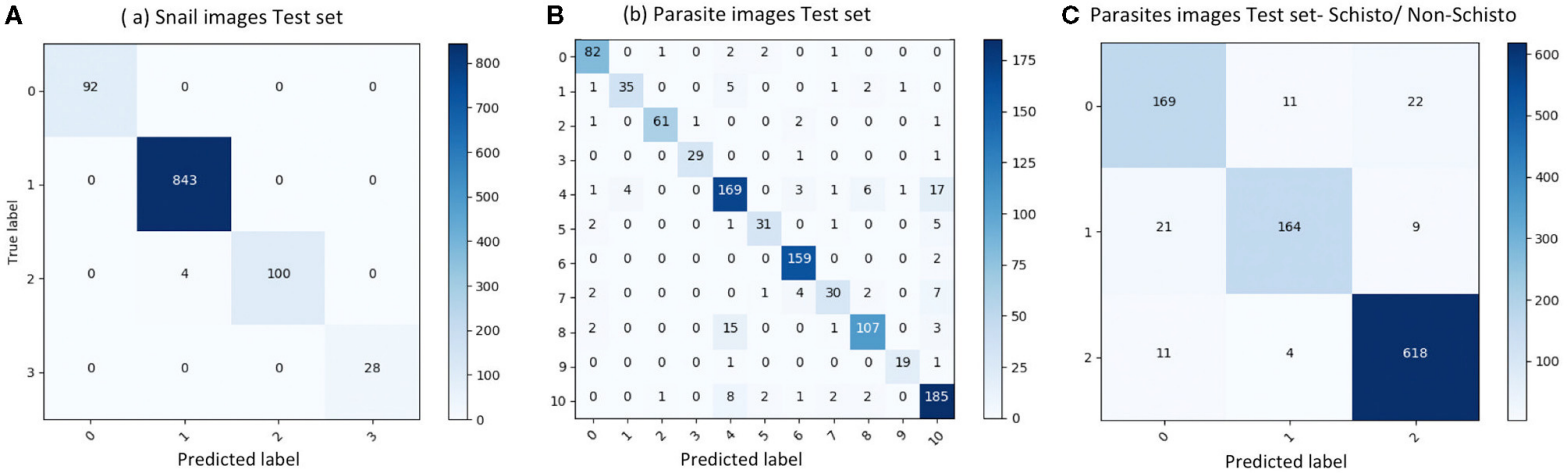
Confusion matrix of classification on test set. **(A)** results of snail image set, labels: 0-*Biomphalaria* spp., 1: *Bulinus* spp., 2: *Radix natalensis*, 3: *Melanoides* spp. **(B)** results of parasite image set, labels: 0: Amphistome cercariae, 1: *Schistosoma bovis*, 2: Echinostome cercariae, 3: Gymnocephalus cercariae, 4: Human-schisto, 5: Metacercaria, 6: Parapleurolophocercous cercariae, 7: Parthenitae, 8: Non-human- schisto forktail type I, 9: Non-human- schisto forktail type II, 10: Xiphidiocercariae. **(C)** Combining other trematodes as one category, labels: 0: Schisto, 1: Non-human forktail type I, type II, and *bovis*, 2: Other trematodes.

We evaluated our CNN model's performance with metrics of accuracy, sensitivity, specificity, and F1 score on the validation set. With the optimized CNN architecture and hyperparameters, we obtained 99.60% accuracy with VGG16 (proportion of correct classifications, either true positive or true negative) for the 4 snail genera and 91.21% accuracy with InceptionResNet V2 for the 11 parasite morphotypes. The optimized dropout rates for the convolutional layer (21) for the snail dataset was 0.6 and for parasite dataset, 0.45. For the parasite set, we ran a second analysis with only three categories of cercariae relevant to map risk for schistosomiasis transmission, namely: human schistosomes, non-human forktail cercariae, and other trematode morphotypes. The overall accuracy for the three parasite categories increased to 94.78%. The sensitivity of distinguishing human schistosomes from non-human cercaria was 85.00%. Figure 3 shows the confusion matrix of our method over the four snail genera and 11 parasite morphotypes, along with details of true positive (TP), true negative (TN), false positive (FP), and false negative (FN) outcomes.

Sensitivity, specificity, recall, precision, and F1 score for each categories were calculated as follows:

(1) sensitivity=recall=TP/(TP+FN)

(2) specificity=TN/(TN+FP)

(3) precision=TP/(TP+FP)

(4) $$\text{F}1\text{ score}={\left(\frac{recall^{-1}+\;precision^{-1}}{2}\right)}^{-1}$$

Sensitivity measures the proportion of positives that are correctly identified; in our case, we focused on the percentage of human schistosomes correctly identified. Specificity measures the proportion of negatives that are correctly identified; we focused on the percentage of non-human fork-tailed cercariae (some of which can potentially be visually similar and hard to distinguish from human schistosomes) that were correctly identified as non-human schistosomes. Precision is a measure of a classifier's exactness, while recall is a measure of a classifier's completeness. Low precision indicates many false positives, while low recall indicates a large number of false negatives. The F1 score conveys the balance between precision and recall, defined as the harmonic mean of precision and sensitivity. Our results showed that the CNN produced high sensitivity and high specificity, as well as an acceptable F1 score in all categories (Table 2). The metrics to measure classification performances are shown in Table 2 and demonstrate the robustness of the CNN training algorithm for the tasks of image recognition for both snails and cercaria, despite the low sample size. The details of all the training experiments with seven selected pre-trained models, as well as the confusion matrix, are provided in the supplemental materials (see Supplementary Appendix 3); only the best model statistics and metrics (VGG16 for snail dataset and InceptionResNetV2 for parasite dataset) are reported in the result section.

### Comparison With Human Parasitologist Performance

**Figure 4:**
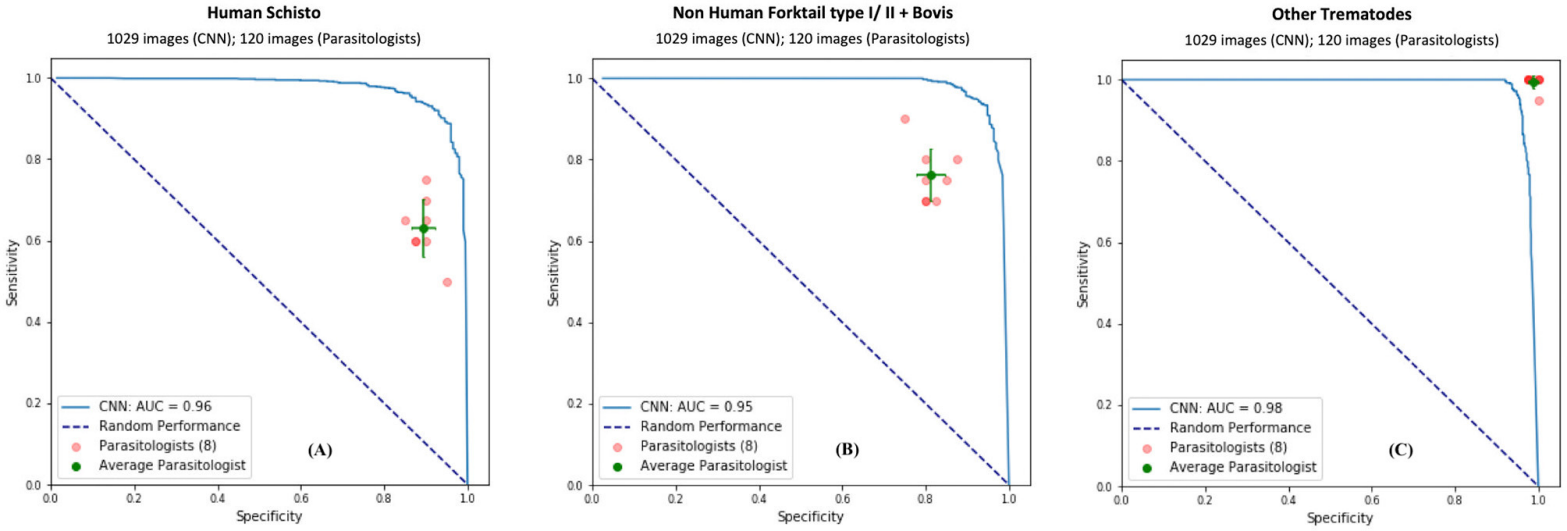
Comparison of classification performance with CNN and parasitologist. CNN's performance represented by the ROC curve (in blue) exceeds that of trained parasitologists when their sensitivity and specificity points (in red) fall below the ROC curve. The green points represent the average of the parasitologists (average sensitivity and specificity of all red points), with error bars denoting one standard deviation. We simplify the 11 categories of parasite to only three categories of interest for schistosomiasis environmental risk mapping: **(A)** human schistosomes, **(B)** non-human forktail cercariae, and **(C)** other trematode morphotypes. The area under the curve (AUC) for each case is over 95%.

To validate our deep learning approach, we compared the direct performance of the CNN to eight trematode parasitology experts. For each image, the parasitologists were asked to identify the category of the snails and cercaria from single images. We prepared 30 snail images from among the four categories and 120 parasite images from among the 11 morphotypes in the CNN's hold-out validation sets. For each test, previously unseen, molecularly-verified images of trematode cercariae and snails were displayed, and parasitologists were asked to identify them from among the same categories of snails and cercaria on which the computer vision algorithm trained. The parasitologists were provided a standardized identification guideline, and key, along with the quiz. A sample quiz is included in supplement materials (see Supplementary Appendix 4). The metrics used to measure human parasitologists' performances and compare with the CNN's, such as sensitivity, specificity, rand F1 score are shown in Table 3 and Figure 4. The CNN generated a malignancy probability *P* per image. We then fixed a threshold probability *t* such that the prediction ŷ for any image is ŷ* if P* ≥ *t*, and the Receiver Operating Characteristic (ROC) curve (blue line in Figure 4) is drawn by sweeping *t* in the interval 0 to 1 (36). The area under the curve (AUC) is the CNN's measure of performance, with a maximum value of 1. The AUC for human schistosomes, non-human fork-tailed cercariae, and other trematodes were 0.96, 0.95, 0.98, respectively. The CNN's classification performance matched, or was slightly superior to, that of trained parasitologists in the case of schistosome/fork-tail parasites, whereas, it was slightly inferior in the case of non-fork-tail parasites.

**Table 3:** Results of the identification of snails and cercaria by eight parasitologists.

	**TP**	**TN**	**FP**	**FN**	**sensitivity(%)**	**specificity(%)**	**F1 score**
**Snail category**							
*Biomphalaria*	2	8	0	0	100.00	100.00	1.00
*Bulinus*	3	7	0	0	100.00	100.00	1.00
*Radix natalensis*	3	7	0	0	100.00	100.00	1.00
*Melanoides spp*.	2	8	0	0	100.00	100.00	1.00
**Parasite category**							
Schisto	15	99	5	1	93.75	95.19	0.83
Non-human forktail	19	95	3	5	79.17	96.94	0.83
Other trematodes	78	40	0	2	97.50	100.00	0.98

*TP, true positive; TN, true negative; FP, false positive; FN, false negative*.

## Discussion

Here we presented a machine learning model that was capable of accurately classifying images of larval human schistosome cercariae and their intermediate host snails. Our CNN classification performed significantly better for snail recognition (average accuracy: 99.33%) than for parasite recognition (average accuracy: 91.78%). This is because snail morphologies are generally more distinct, and it is easier to take high quality pictures of snails using a wide variety of cameras (including cellular phones) than that for cercariae, which require photography through an optical microscope. Better imagery and very distinct morphological features between competent *Bulinus* and *Biomphalaria* snails and non-competent snail species decreased erroneous misclassification of snails, as reflected by the small number of false positives and false negatives in our model evaluation. Cercariae, on the other hand, have complex morphologies, with many more categories defined in our dataset, and are usually moving at the time of the image capture, which all makes discrimination of trematode cercariae more challenging, as reflected by the larger number of false negative and false positive classifications.

Despite these limitations, our analysis showed that our CNN can be trained with rather limited image datasets using data augmentation approaches. Given the small size of our dataset for morphologically similar forked-tail human and non-human schistosome cercariae, we used rotation and shifting methods to effectively increase the number of images used in training (but we avoided mirror image augmentation to preserve the ability of the algorithm to distinguish sinistral vs. dextral coiling in different snail genera). In addition to thousands of still images, we had the advantage of access to some limited video footage of living, mobile cercariae, which allowed us to generate additional still-images of cercariae and to increase samples for training. Although, we only used video to augment still-images to our training data, video could be explored in the future as an input to a new classification algorithm, one which also considers swimming patterns that may help in distinguishing cercariae to species (37).

In addition to building a deep learning model, we also developed a workflow for image collection and quality control as a guideline for other researchers interested in using this tool. This would allow anyone to quickly build an image database that can facilitate the identification of medically important snails and their cercariae with the click of a cell phone and a pre-trained computer vision model. A protocol for image collection is provided in the supplemental materials (see Supplementary Appendix 2). We hope that as this protocol is adopted, user photos can be aggregated into a global database of medically important snail and parasite images, including snail and parasite species that are not relevant to human health but will be useful to improve model training. When collecting the samples in the field, we also suggest that GPS locations be recorded, which can facilitate precision-mapping of medically important snail and parasite distributions, over space and time, and across broad and variable geographies and contexts. For all of these activities, we take inspiration from online platforms that encourage citizen science and crowdsourced data, including iNaturalist (38), NaturNet (39), and MycoMap iNaturalist (40), for example, has the image classification capacity to immediately suggest an object name right after a user has uploaded an image to the platform.

The CNN algorithm was trained and evaluated on snails from the lower basin of the Senegal river, a fairly limited geographical area encompassing only a small fraction of a wider mollusk and trematode biodiversity. In the near future, the goal is to expand the analysis to other geographical areas in Africa and Latin America where schistosomiasis and other gastropod-borne helminthiasis (41) are endemic, as well as to a wider range of habitats, from natural to human dominated environments, where snails of medical importance thrive. At present, only the database from the lower basin of the Senegal river had the number of digitized snails and parasites images and the level of classification accuracy required to train a convolutional neural network, and, as such, our study represents a proof-of-concept. However, we envision that imagery of snail internal organs could supplement shell photos in our future work with machine learning, whenever internal morphological differences might aid in species identification. We expect that increasing the training dataset and gathering imagery from more locations will support the improvement of our CNN model accuracy and its potential use in broader geographical areas. In addition, *Biomphalaria* species in the Americas have much greater diversity than in Africa, and snails that can host parasites of medical importance can be more challenging to identify morphologically through shell morphology alone (2) which limits the ability of CNNs based on shell photographs to identify snails lower than at the level of genus on that continent.

To encourage the development of our specific platform, and for translating it for use on other medically relevant cercariae, vectors, and non-human hosts found in the environment, our code, image sets, and neural network weights are provided in a public repository. This includes detailed documentation to assist researchers in following our workflow to (1) reproduce the results, and (2) build new models using their own image datasets. We have also deployed our classification model to a web application that allows researchers to select their own images of snails and cercariae to obtain the classification prediction, which is the probability of an object belonging to a specific category. Despite the requirement of considerable computing power to properly train a CNN image classification model, once trained and deployed, a CNN can provide a result – in a fraction of a second – on a smartphone and a common laptop computer and can also express clear indicators of uncertainty along with the classification, which can also be useful to scientists, citizens, and decision-makers.

This proof-of-concept study was intended to show that CNN can classify snails and cercariae with a reasonable accuracy even on datasets of limited size. Its accuracy has great potential to improve in the future, with more high-quality imagery of snails and their respective cercariae being made available by the scientific and citizen-science communities.

However, our work does not dismiss the continued relevance of classic parasitology training. Our model is not designed to be a substitute for experienced parasitologists in pertinent scientific field studies, but as a tool to assist researchers in resource-limited settings where trained parasitologists may not be available to perform regular schistosomiasis risk assessments. In the spirit of other citizen science applications, we hope that our CNN web application will also foster interest in exploring parasite biodiversity, as well as increase awareness of schistosomiasis transmission caused by the presence of snails of medical importance. For example, we envision our CNN application could be used in K-12 and college education, to inspire a new generation of scientists to leverage new and affordable technologies to support the crucial work of global infectious disease environmental diagnostics and sustainable control.

## Conclusion

This study demonstrates the effectiveness of deep learning in image recognition tasks for classification of medically relevant snails and their parasite counterparts from the Senegal River in West Africa. We apply a computer vision model, using a single convolutional neural network trained on a few thousand images, facilitated by transfer learning and pre-trained models. We present a proof-of-concept model for this technology to be further honed and someday applied in resource-poor settings where schistosomiasis is endemic and where the identification of hotspots of transmission is desperately needed to target interventions. The performance of our model was comparable to that of eight highly trained human parasitologists who were all familiar with snail and parasite diversity in the study region, but within a controlled test setting and with a specific and highly curated set of snail and parasite species. In light of our promising results, we have deployed our product as a publicly accessible web application as an exploratory and educational use case. In the future, this method could be deployed on mobile devices with minimal cost and holds the potential for substantial improvement for monitoring and identifying snail and schistosomiasis hotspots (42). Deep learning is a powerful tool that can help fill the gap that currently limits our understanding of the environmental components of transmission for a variety of neglected tropical diseases.

## Data Availability Statement

The raw data supporting the conclusions of this article will be made available by the authors, without undue reservation.

## Author Contributions

ZL, AC, and KT built, trained, and validated the CNN model. AC, IJ, and SS organized and labeled the image dataset. AC, IJ, SS, GR, NJ, RN, LB, and GD conducted the field work. PS performed the initial training and testing. KT found the model with highest accuracy and further improved the model. PE and TN deployed the CNN model and created the web application. KT, GD, ZL, AC, SS, and IJ edited and revised the manuscript. GD conceived the idea and supervised the project in all perspectives. All authors contributed to the article and approved the submitted version.

## Conflict of Interest

PE and TN were employed by the company IBM Silicon Valley Lab, San Jose, CA 95141, USA. The remaining authors declare that the research was conducted in the absence of any commercial or financial relationships that could be construed as a potential conflict of interest.

## Supplementary Material

Supplementary Appendix 1 Protocols for image collection and quality control.

Supplementary Appendix 2 Field ID guidelines for morphologies of snails and cercariae.

Supplementary Appendix 3 CNN metrics and performance details for the 7 selected pre-trained models.

Supplementary Appendix 4 Sample quiz on snail and parasite classification for human parasitologists.

## References

[1] Sokolow SH Jones IJ Jocque M La D Cords O Knight A. Nearly 400 million people are at higher risk of schistosomiasis because dams block the migration of snail-eating river prawns. Philos Trans R Soc Lond B Biol Sci. (2017) 372:20160127. 10.1098/rstb.2016.0127 28438916 PMC5413875

[2] Sokolow SH Wood CL Jones IJ Swartz SJ Lopez M Hsieh MH. Global assessment of schistosomiasis control over the past century shows targeting the snail intermediate host works best. PLoS Negl Trop Dis. (2016) 10:e0004794. 10.1371/journal.pntd.0004794 27441556 PMC4956325

[3] WHO. Schistosomiasis: Number of People Treated Worldwide in 2014. WHO. Available online at: http://www.who.int/schistosomiasis/resources/who_wer9105/en/ (accessed March 18, 2021).

[4] Steinmann P Keiser J Bos R Tanner M Utzinger J. Schistosomiasis and water resources development: systematic review, meta-analysis, and estimates of people at risk. Lancet Infect Dis. (2006) 6:411–25. 10.1016/S1473-3099(06)70521-7 16790382

[5] Laidemitt MR Anderson LC Wearing HJ Mutuku MW Mkoji GM Loker ES. Antagonism between parasites within snail hosts impacts the transmission of human schistosomiasis. ELife. (2019) 8:e50095. 10.7554/eLife.50095 31845890 PMC6917487

[6] Sokolow SH Huttinger E Jouanard N Hsieh MH Lafferty KD Kuris AM. Reduced transmission of human schistosomiasis after restoration of a native river prawn that preys on the snail intermediate host. Proc Natl Acad Sci USA. (2015) 112:9650–5. 10.1073/pnas.1502651112 26195752 PMC4534245

[7] Mungai PL Muchiri EM King CH Abbasi I Hamburger J Kariuki C. Differentiating Schistosoma haematobium from related animal Schistosomes by PCR amplifying inter-repeat sequences flanking newly selected repeated sequences. Am J Trop Med Hyg. (2012) 87:1059–64. 10.4269/ajtmh.2012.12-0243 23109375 PMC3516075

[8] Rajkomar A Hardt M Howell MD Corrado G Chin MH. Ensuring fairness in machine learning to advance health equity. Ann Intern Med. (2018) 169:866–72. 10.7326/M18-1990 30508424 PMC6594166

[9] Jaiswal AK Tiwari P Kumar S Gupta D Khanna A Rodrigues JJPC. Identifying Pneumonia in Chest X-rays: A Deep Learning Approach. Available online at: https://uobrep.openrepository.com/handle/10547/623797 (accessed March 17, 2021).

[10] Ker J Wang L Rao J Lim T. Deep learning applications in medical image analysis. IEEE Access. (2018) 6:9375–89. 10.1109/ACCESS.2017.2788044

[11] Wood CL Sokolow SH Jones IJ Chamberlin AJ Lafferty KD Kuris AM. Precision mapping of snail habitat provides a powerful indicator of human schistosomiasis transmission. Proc Natl Acad Sci USA. (2019) 116:23182–91. 10.1073/pnas.1903698116 31659025 PMC6859407

[12] Aksenova OV Bolotov IN Gofarov MYu Kondakov AV Vinarski MV Bespalaya YV. Species richness, molecular taxonomy and biogeography of the radicine pond snails (gastropoda: lymnaeidae) in the old world. Sci Rep. (2018) 8:11199. 10.1038/s41598-018-29451-1 30046044 PMC6060155

[13] Webster BL Rabone M Pennance T Emery AM Allan F Gouvras A. Development of novel multiplex microsatellite polymerase chain reactions to enable high-throughput population genetic studies of Schistosoma haematobium. Parasit Vectors. (2015) 8:519. 10.1186/s13071-015-1134-5 26329827 PMC4557312

[14] Emery AM Allan FE Rabone ME Rollinson D. Schistosomiasis collection at NHM (SCAN). Parasit Vectors. (2012) 5:185. 10.1186/1756-3305-5-185 22943137 PMC3453491

[15] Folmer O Black M Hoeh W Lutz R Vrijenhoek R. DNA primers for amplification of mitochondrial cytochrome c oxidase subunit I from diverse metazoan invertebrates. Mol Mar Biol Biotechnol. (1994) 3:294–9. 7881515

[16] Kane RA Stothard JR Emery AM Rollinson D. Molecular characterization of freshwater snails in the genus Bulinus: a role for barcodes? Parasit Vectors. (2008) 1:15. 10.1186/1756-3305-1-15 18544153 PMC2441610

[17] Russakovsky O Deng J Su H Krause J Satheesh S Ma S. Imagenet large scale visual recognition challenge. Int J Comput Vis. (2015) 115:211–52. 10.1007/s11263-015-0816-y

[18] Araújo T Aresta G Castro E Rouco J Aguiar P Eloy C. Classification of breast cancer histology images using Convolutional Neural Networks. PLoS ONE. (2017) 12:e0177544. 10.1371/journal.pone.0177544 28570557 PMC5453426

[19] Esteva A Kuprel B Novoa RA Ko J Swetter SM Blau HM. Dermatologist-level classification of skin cancer with deep neural networks. Nature. (2017) 542:115–8. 10.1038/nature21056 28117445 PMC8382232

[20] LeCun Y Bengio Y. Convolutional networks for images, speech, and time series. Handb Brain Theory Neural Netw. (1997) 3361:3–11.

[21] Spanhol FA Oliveira LS Petitjean C Heutte L. Breast cancer histopathological image classification using Convolutional Neural Networks. In: 2016 International Joint Conference on Neural Networks (IJCNN). Vancouver, BC: IEEE (2016). p. 2560–7. 10.1109/IJCNN.2016.7727519

[22] Pan SJ Yang Q. A survey on transfer learning. IEEE Trans Knowl Data Eng. (2010) 22:1345–59. 10.1109/TKDE.2009.191

[23] Bahadori MT Liu Y Zhang D. Learning with minimum supervision: a general framework for transductive transfer learning. In: 2011 IEEE 11th International Conference on Data Mining. Vancouver, BC: IEEE. (2011). p. 61–70. 10.1109/ICDM.2011.92

[24] Simonyan K Zisserman A. Very deep convolutional networks for large-scale image recognition. CoRR abs/1409.1556 (2015).

[25] Szegedy C Vanhoucke V Ioffe S Shlens J Wojna Z. Rethinking the inception architecture for computer vision. In: 2016 IEEE Conference on Computer Vision and Pattern Recognition (CVPR). Las Vegas, NV: IEEE (2016). p. 2818–26. 10.1109/CVPR.2016.308

[26] Chollet F. Xception: Deep Learning with Depthwise Separable Convolutions. (2016). Available online at: https://arxiv.org/abs/1610.02357v3 (accessed March 17, 2021).

[27] He K Zhang X Ren S Sun J. Deep Residual Learning for Image Recognition. (2015). Available online at: https://arxiv.org/abs/1512.03385v1 (accessed March 17, 2021).

[28] Szegedy C Ioffe S Vanhoucke V Alemi A. Inception-v4, Inception-ResNet and the Impact of Residual Connections on Learning. (2016) Available online at: https://arxiv.org/abs/1602.07261v2 (accessed March 17, 2021).

[29] LeCun Y Bengio Y Hinton G. Deep learning. Nature. (2015) 521:436–44. 10.1038/nature14539 26017442

[30] Nair V Hinton GE. Rectified linear units improve restricted boltzmann machines. In: Proceedings of the 27th International Conference on International Conference on Machine Learning ICML'10. Madison, WI: Omnipress (2006). p. 807–14.

[31] keras-team/keras. Keras. (2021). Available online at: https://github.com/keras-team/keras (accessed March 17, 2021).

[32] Abadi M Barham P Chen J Chen Z Davis A Dean J. TensorFlow: A System for Large-Scale Machine Learning. Usenix (2016).

[33] Dosovitskiy A Springenberg JT Brox T. Unsupervised Feature Learning by Augmenting Single Images. (2013). Available online at: https://arxiv.org/abs/1312.5242v3 (accessed March 17, 2021).

[34] Courbariaux M Bengio Y David J-P. Binary Connect: Training Deep Neural Networks with Binary Weights during Propagations. (2015). Available online at: https://arxiv.org/abs/1511.00363v3 (accessed March 17, 2021).

[35] Smilkov D Thorat N Assogba Y Yuan A Kreeger N Yu P. TensorFlow.js: Machine Learning for the Web and Beyond. (2019) Available online at: https://arxiv.org/abs/1901.05350v2 (accessed March 17, 2021).

[36] Florkowski CM. Sensitivity, specificity, receiver-operating characteristic (ROC) curves and likelihood ratios: communicating the performance of diagnostic tests. Clin Biochem Rev. (2008) 29 (Suppl. 1):S83–7. 18852864 PMC2556590

[37] Weinstein BG. Scene-specific convolutional neural networks for video-based biodiversity detection. Methods Ecol Evol. (2018) 9:1435–41. 10.1111/2041-210X.13011

[38] Wittmann J Girman D Crocker D. Using iNaturalist in a Coverboard Protocol to Measure Data Quality: Suggestions for Project Design. Available online at: https://theoryandpractice.citizenscienceassociation.org/articles/10.5334/cstp.131/ (accessed March 18, 2021).

[39] Stracke CM. Competence modelling for innovations and quality development in E-Learning: towards learning outcome orientation by competence models. In: Proceedings of World Conference on Educational Multimedia, Hypermedia and Telecommunication 2011. Chesapeake, VA: AACE (2011). p. 1885–94.

[40] MycoMap Lac Qui Parle. iNaturalist. Available online at: https://www.inaturalist.org/projects/mycomap-lac-qui-parle (accessed March 18, 2021).

[41] Giannelli A Cantacessi C Colella V Dantas-Torres F Otranto D. Gastropod-borne helminths: a look at the snail-parasite interplay. Trends Parasitol. (2016) 32:255–64. 10.1016/j.pt.2015.12.002 26740470

[42] Holmström O Linder N Ngasala B Mårtensson A Linder E Lundin M. Point-of-care mobile digital microscopy and deep learning for the detection of soil-transmitted helminths and *Schistosoma haematobium*. Glob Health Action. (2017) 10:1337325. 10.1080/16549716.2017.1337325 28838305 PMC5645671

[43] deleo-lab/schisto-parasite-classification. De Leo Lab. (2019). Available online at: https://github.com/deleo-lab/schisto-parasite-classification (accessed March 18, 2021).

