# Supplementary material for: Identification of Snails and Schistosoma of Medical Importance via Convolutional Neural Networks: A Proof-of-Concept Application for Human Schistosomiasis
Source: Front Public Health. 2021 Jul 15;9:642895. doi: 10.3389/fpubh.2021.642895 (PMC8319642; doi:10.3389/fpubh.2021.642895)
Supplement: Supplementary Appendix 1 — Protocols for image collection and quality control. [file Data_Sheet_1.pdf]

Supplementary Figure

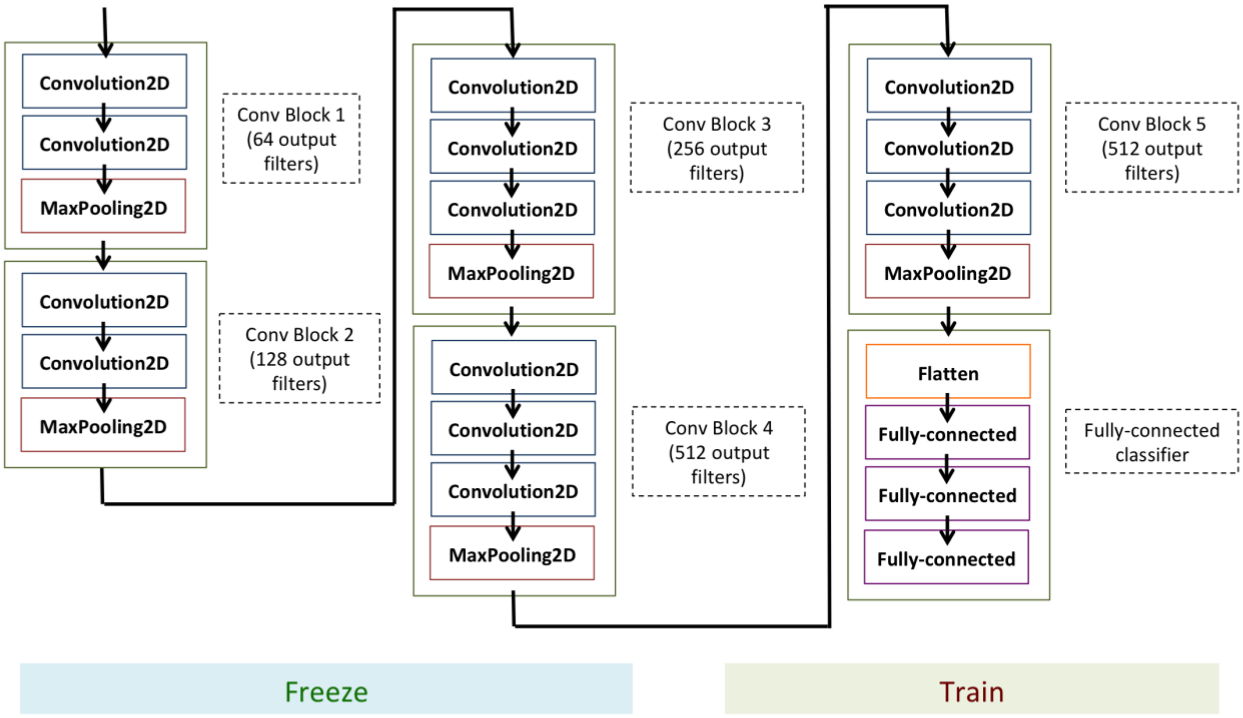

Figure S1: VGG-16 Architecture

Supplementary Appendix

Supplementary Appendix 1 | Protocols for image collection and quality control.

Protocol description for gathering pictures of snails/parasites

Field Collection:

When collecting snails in the field, there was a broad habitat type from which the snails were recovered.

Take careful note of which sampling point each snail came from, and clearly label the collection containers by distinct sampling point locations.

Record GPS coordinates of the water point where sampling is occurring using a cell phone GPS receiver. If a higher accuracy GPS device available, take the GPS coordinates of the individual sampling points where snails were recovered.

If the snails were recovered in vegetation, take note of what category of vegetation – tall emergent reeds, shorter grasses/sedges, or floating vegetation. Record the vegetation species if known; otherwise describe it in as best detail as possible, using pictures if a camera is available. This will facilitate consistency of vegetation/habitat description across different sites and time points and may allow for later identification of unknown vegetation species.

If snails were collected using a timed search method, data gathered thus far are sufficient.

If snails were collected via exhaustive sampling in a defined unit of area, such as quadrat, remove all vegetation from sample area/quadrat, remove all snails from that vegetation, shake the water off and record the mass of vegetation. If many vegetation species are present in the quadrat, separate the species and weigh separately. These data are useful in understanding the fine-scale distribution and ecology of these snails.

Lab processing:

The number of images necessary to train a model to distinguish snail categories will depend on the amount of variation both within and between snail shell morphologies. For our model, we attained good performance using a few hundred images of relevant snail species. If the snail species of interest are visually very similar, more pictures will need to be gathered to ensure good model performance. Photographing snails is best accomplished in an indoor laboratory setting after collecting snails from the field, because maintaining consistency across images is generally more feasible indoors than outdoors. When processing the snails in the lab, clearly label the collection point of each snail. While the snail is still intact, place it on a petri dish under a dissecting microscope, set a scale bar next to it, and photograph both sides of the snail shell through the microscope eyepiece or the trinocular port, if available. Keeping the camera mounted to either one of the eyepieces or the trinocular port is ideal for consistency; however, with a little practice it is possible to hold a basic digital camera or cell phone to the eyepiece and quickly gather pictures that way. One picture (from each side) of each individual snail is enough, and more than one may cause the training data to become biased toward that one individual's appearance. The classification model is susceptible to confounding factors when learning, so reducing variation of image background characteristics will help improve model performance. Try to maximize uniformity for all images by keeping the lighting of the room and of the microscope consistent, using the same camera and camera settings, and using the same magnification settings. Maintaining consistency of background image conditions will allow the model to learn more about the features of the snails, and not attempt to find spurious associations between images. For example, if for one week the microscope lighting conditions are 50% lower brightness than the following week, the model may assign those snails to two categories: brightly lit snails and dimly lit snails.

Any species that cannot be visually identified by lab technicians with complete certainty will require molecular identification to avoid training the model on incorrectly classified images. For

this process, we removed the hepatopancreas before vouchering the snails, because that organ can releasee digestive enzymes that may degrade DNA. The remaining snail tissue was then vouchered in 95% ethanol in a 1.5 mL microcentrifuge tube, sealed with parafilm, and shipped to a facility with expertise in molecular analysis of snails from this region. Each individual snail voucher vial ought to be clearly labeled using waterproof paper and pencil using unique ID's that allow researchers to clearly know when and where each individual snail came from – this will facilitate the connection of snail images to molecular identification results.

The protocol for building a machine learning model for identifying parasites is largely the same as for snails. However, instead of attempting to photograph the cercariae through the dissecting scope, it is better to make slides of these organisms and take photographs on a compound microscope using 100x magnification. Many pictures must be gathered, and DNA samples will likely be necessary as well in areas of high cryptic species diversity. Cercarial DNA samples are easy to collect and store on Whatman Indicating FTA cards – one single card can store upwards of 100 individual cercarial samples, using 3-4 µl of water per aliquot using a micropipette. While the snails are usually identified using shell features and therefore should only require a maximum of two photos per snail, cercaria are highly dynamic organisms, often moving rapidly and changing their morphology constantly as they expand and contract their bodies in order to swim through the water. It is therefore useful to take many pictures of cercaria as they swim to capture the range of morphology they can exhibit. However, it is not recommended to overbalance the image dataset by having too many images of an individual cercaria, so limit the number of images of each individual. Move the slides around and attempt to capture as much variation between individuals as possible. Use the same recommendations for best practices for collection of these images as explained previously regarding collection of snail images – uniformity of background light intensity, color, and contrast are even more important with these highly dynamic organisms.

Supplementary Appendix 2 | Field ID guidelines for morphologies of snails and cercariae.

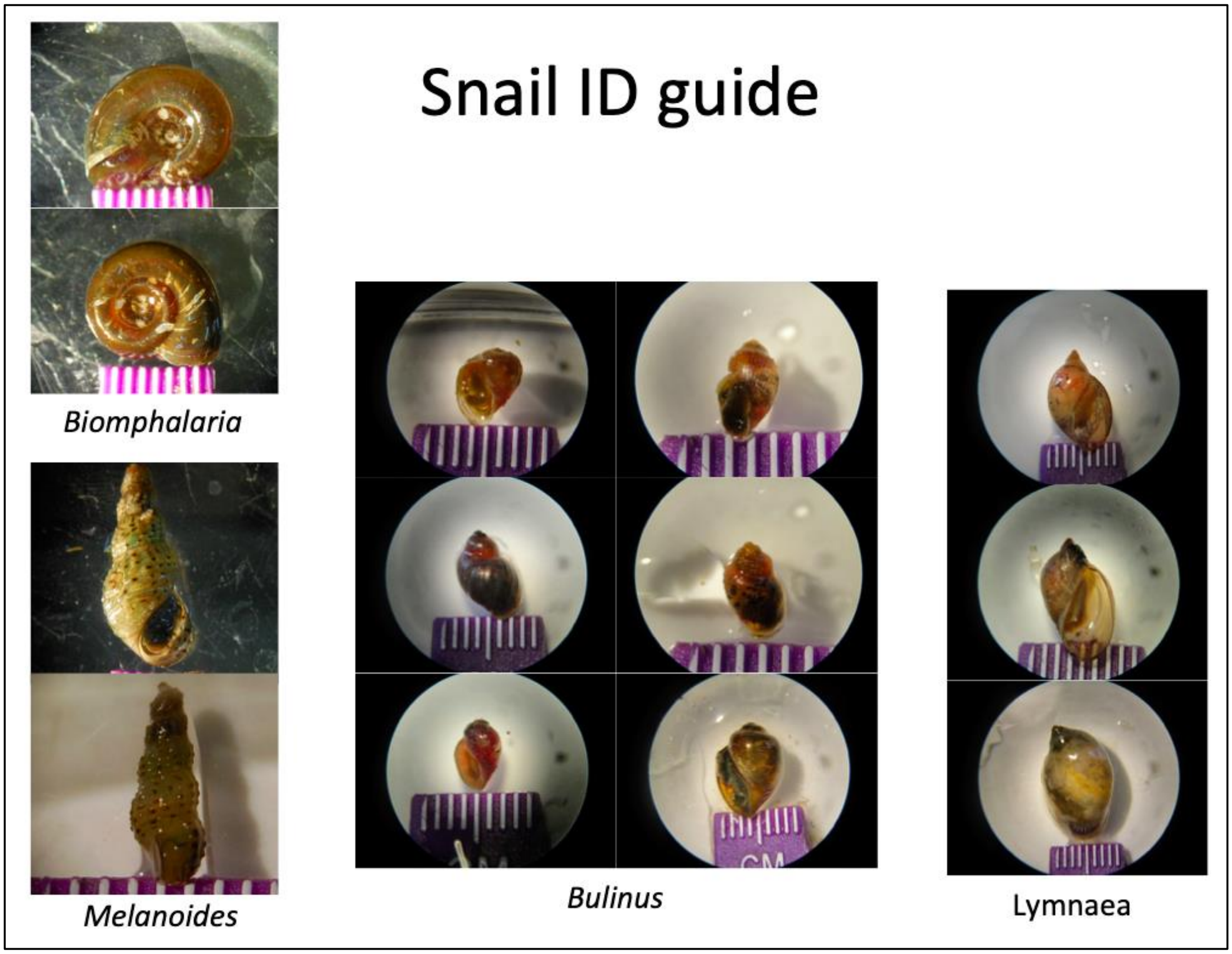

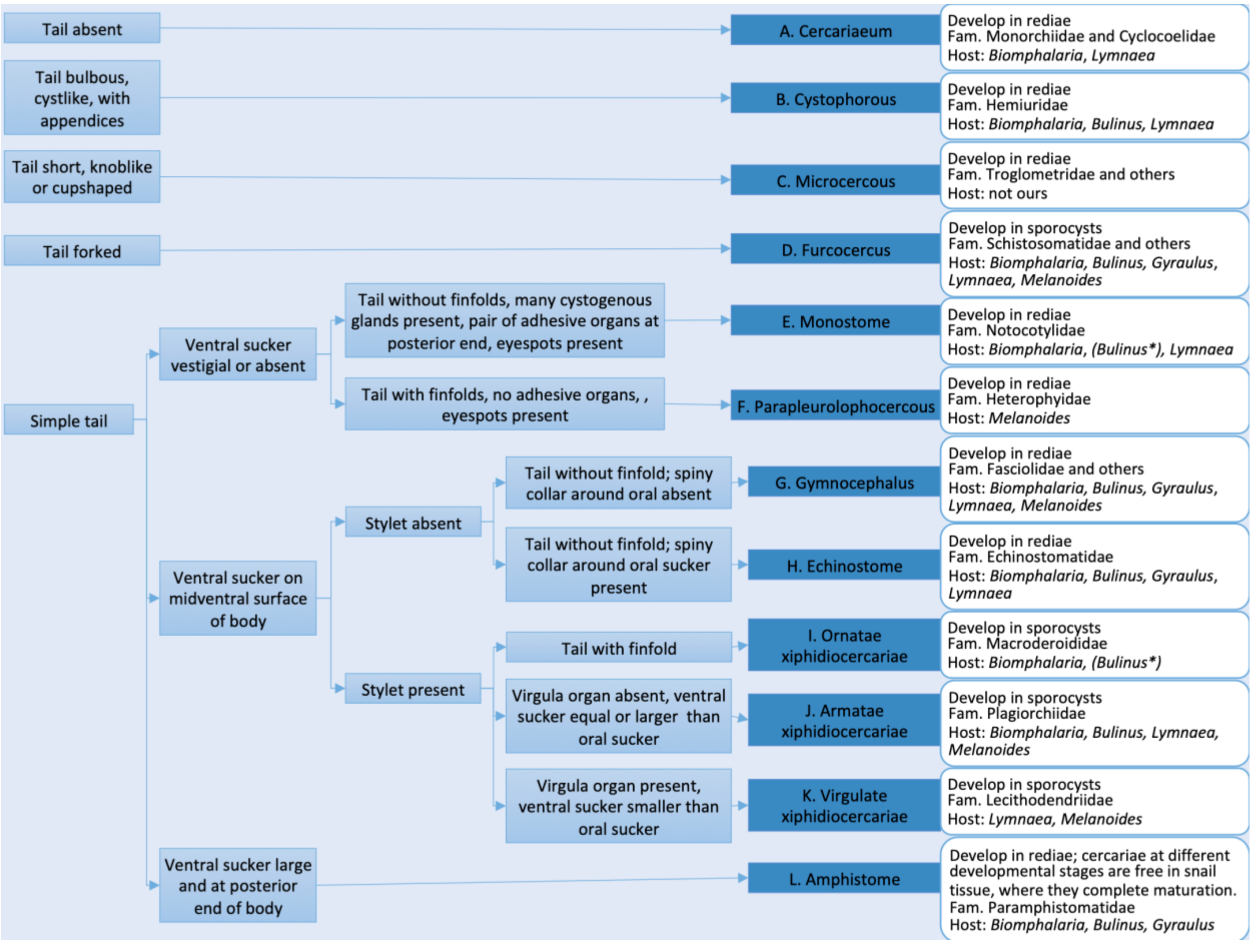
Tail absent
A. Cercariaeum
Develop in rediae
Fam. Monorchiidae and Cyclocoelidae
Host: *Biomphalaria, Lymnaea*
Tail bulbous, cystlike, with appendices
B. Cystophorous
Develop in rediae
Fam. Hemiuridae
Host: *Biomphalaria, Bulinus, Lymnaea*
Tail short, knoblike or cupshaped
C. Microcercous
Develop in rediae
Fam. Troglometridae and others
Host: not ours
Tail forked
D. Furcocercus
Develop in sporocysts
Fam. Schistosomatidae and others
Host: *Biomphalaria, Bulinus, Gyraulus, Lymnaea, Melanoides*
Simple tail
Ventral sucker vestigial or absent
Tail without finfolds, many cystogenous glands present, pair of adhesive organs at posterior end, eyespots present
E. Monostome
Develop in rediae
Fam. Notocotylidae
Host: *Biomphalaria, (Bulinus*), Lymnaea*
Tail with finfolds, no adhesive organs, , eyespots present
F. Parapleurolophocercous
Develop in rediae
Fam. Heterophyidae
Host: *Melanoides*
Ventral sucker on midventral surface of body
Stylet absent
Tail without finfold; spiny collar around oral absent
G. Gymnocephalus
Develop in rediae
Fam. Fasciolidae and others
Host: *Biomphalaria, Bulinus, Gyraulus, Lymnaea, Melanoides*
Tail without finfold; spiny collar around oral sucker present
H. Echinostome
Develop in rediae
Fam. Echinostomatidae
Host: *Biomphalaria, Bulinus, Gyraulus, Lymnaea*
Stylet present
Tail with finfold
I. Ornatae xiphidiocercariae
Develop in sporocysts
Fam. Macroderoididae
Host: *Biomphalaria, (Bulinus*)*
Virgula organ absent, ventral sucker equal or larger than oral sucker
J. Armatae xiphidiocercariae
Develop in sporocysts
Fam. Plagiorchiidae
Host: *Biomphalaria, Bulinus, Lymnaea, Melanoides*
Virgula organ present, ventral sucker smaller than oral sucker
K. Virgulate xiphidiocercariae
Develop in sporocysts
Fam. Lecithodendriidae
Host: *Lymnaea, Melanoides*
Ventral sucker large and at posterior end of body
L. Amphistome
Develop in rediae; cercariae at different developmental stages are free in snail tissue, where they complete maturation.
Fam. Paramphistomatidae
Host: *Biomphalaria, Bulinus, Gyraulus*

Supplementary Appendix 3 | CNN metrics and performance details for the 7 selected pre-trained models.

Parasites								Prediction															
			Amphistome	Bovis	Echino	Gymno	HumanSchist	Metacerc	Parapleurolop	Parthenitae	Type1	Type2	Xiphidiocercariae										
		index	0	1	2	3	4	5	6	7	8	9	10	# images in te	TP	TN	FP	FN	Sensitivity	Specificity	precision	F1 score	accuracy
	Amphistome	0	82	0	1	0	2	2	0	1	0	0	0	88	82	932	9	6	0.931818182	0.99043571	0.9010989	0.916201117	0.98542274
	Bovis	1	1	35	0	0	5	0	0	1	2	1	0	45	35	980	4	10	0.777777778	0.99593496	0.8974359	0.833333333	0.98639456
	Echino	2	1	0	61	1	0	0	2	0	0	0	1	66	61	961	2	5	0.924242424	0.99792316	0.96825397	0.945736434	0.99319728
	Gymno	3	0	0	0	29	0	0	1	0	0	0	1	31	29	997	1	2	0.935483871	0.998998	0.96666667	0.950819672	0.99708455
	HumanSchist	4	1	4	0	0	169	0	3	1	6	1	17	202	169	795	32	33	0.836633663	0.96130593	0.84079602	0.838709677	0.93683188
Actual	Metacerc	5	2	0	0	0	1	31	0	1	0	0	5	40	31	984	5	9	0.775	0.99494439	0.86111111	0.815789474	0.98639456
	Parapleurolop	6	0	0	0	0	0	0	159	0	0	0	2	161	159	857	11	2	0.98757764	0.98732719	0.93529412	0.960725076	0.98736638
	Parthenitae	7	2	0	0	0	0	1	4	30	2	0	7	46	30	976	7	16	0.652173913	0.99287894	0.81081081	0.722891566	0.9776482
	Type1	8	2	0	0	0	15	0	0	1	107	0	3	128	107	889	12	21	0.8359375	0.98668147	0.89915966	0.866396761	0.96793003
	Type2	9	0	0	0	0	1	0	0	0	0	19	1	21	19	1006	2	2	0.904761905	0.99801587	0.9047619	0.904761905	0.99611273
	Xiphidiocerca	10	0	0	1	0	8	2	1	2	2	0	185	201	185	791	37	16	0.92039801	0.95531401	0.83333333	0.874704492	0.94849368
			91	39	63	30	201	36	170	37	119	21	222	1029									
																907							
															accuracy	0.88143829							
				Schisto	Non-human f	Other trematode		accuracy			TP	TN	FP	FN	Sensitivity	Specificity	precision	F1 score	accuracy				
			Schisto	169	11	22	202	0.83663366		Schisto	169	795	32	33	0.83663366	0.96130593	0.84079602	0.83870968	0.936831876	1029			
			Non-human f	21	164	9	194	0.84536082		Non-human f	164	820	15	30	0.84536082	0.98203593	0.91620112	0.87935657	0.956268222	1029			
			Other tremat	11	4	618	633	0.97630332		Other tremat	618	365	31	15	0.97630332	0.92171717	0.95223421	0.96411856	0.955296404	1029			
				201	179	649	1029																
							951																
						accuracy	0.92419825																

Parasites								Prediction															
			Amphistome	Bovis	Echino	Gymno	HumanSchist	Metacerc	Parapleurolop	Parthenitae	Type1	Type2	Xiphidiocercariae										
		index	0	1	2	3	4	5	6	7	8	9	10	# images in te	TP	TN	FP	FN	Sensitivity	Specificity	precision	F1 score	accuracy
	Amphistome	0	0	1	2	3	4	5	6	7	8	9	10	55	0	794	45	55	0	0.94636472	0	#DIV/0!	0.88814318
	Bovis	1	0	74	0	1	0	5	4	2	0	0	0	86	74	798	10	12	0.860465116	0.98762376	0.88095238	0.870588235	0.9753915
	Echino	2	1	0	4	0	0	37	0	0	0	4	0	46	4	846	2	42	0.086956522	0.99764151	0.66666667	0.153846154	0.950783
	Gymno	3	2	0	0	57	0	0	0	7	0	0	0	66	57	821	7	9	0.863636364	0.99154589	0.890625	0.876923077	0.98210291
	HumanSchist	4	3	0	0	1	28	0	0	1	0	0	0	33	28	857	4	5	0.848484848	0.99535424	0.875	0.861538462	0.98993289
Actual	Metacerc	5	4	0	0	0	0	165	0	4	0	16	0	189	165	605	100	24	0.873015873	0.85815603	0.62264151	0.726872247	0.86129754
	Parapleurolop	6	5	2	0	0	0	3	32	0	0	0	0	42	32	839	13	10	0.761904762	0.98474178	0.71111111	0.735632184	0.97427293
	Parthenitae	7	6	0	0	1	0	0	0	155	0	1	0	163	155	704	27	8	0.950920245	0.9630643	0.85164835	0.898550725	0.96085011
	Type1	8	7	6	0	0	0	17	3	4	9	3	0	49	9	837	8	40	0.183673469	0.99053254	0.52941176	0.272727273	0.94630872
	Type2	9	8	1	0	1	0	24	0	2	0	99	0	135	99	725	34	36	0.733333333	0.95520422	0.7443609	0.738805970	0.92170022
	Xiphidiocerca	10	9	0	0	0	0	9	0	0	0	1	11	30	11	854	10	19	0.366666667	0.98842593	0.52380952	0.431372549	0.96756152
			45	84	6	64	32	265	45	182	17	133	21	894									
																634							
															accuracy	0.70917226							
				Schisto	Non-human f	Other trematode		accuracy			TP	TN	FP	FN	Sensitivity	Specificity	precision	F1 score	accuracy				
			Schisto	28	0	4	32	0.875		Schisto	28	657	4	4	0.875	0.99394856	0.875	0.875	0.988455988	693			
			Non-human f	0	182	13	195	0.93333333		Non-human f	182	498	0	13	0.93333333	1	1	0.96551724	0.981240981	693			
			Other tremat	4	0	462	466	0.99141631		Other tremat	462	210	17	4	0.99141631	0.92511013	0.96450939	0.97777778	0.96969697	693			
				32	182	479	693																
							672																
						accuracy	0.96969697																

Supplementary Appendix 4 | Sample quiz on snail and parasite classification for human parasitologists

Snail ID quiz v2

* Required

Snail ID quiz v2

Which snail is this? * 1 point

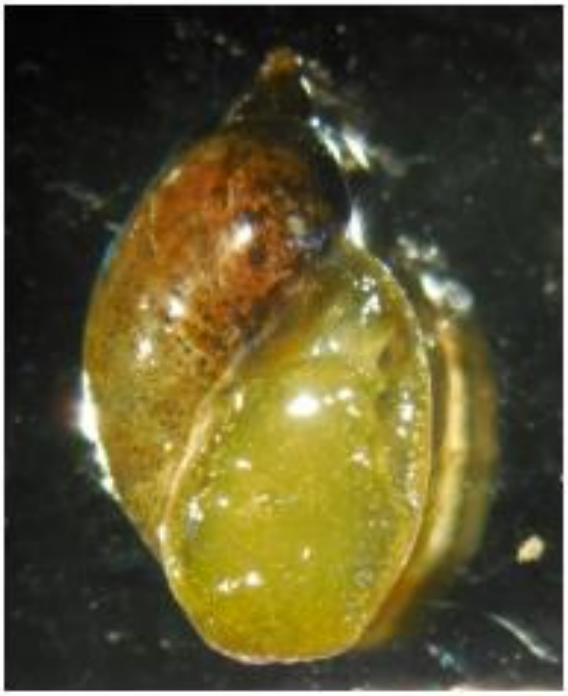

- Biomphalaria
- Bulinus
- Lymnaea
- Melanoides

Trematode Cercariae ID quiz v1

* Required

Trematode Cercariae ID quiz v1

Which category does this image fit into? 1 point
*

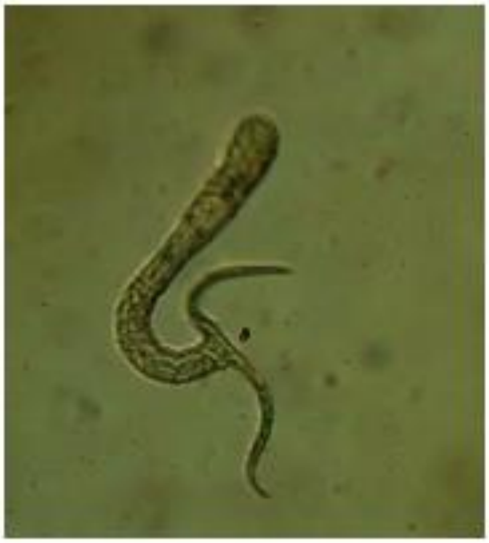

- Amphistome cercaria
- Parapleurolophocercous cercaria
- Human schistosome cercaria
- Metacercaria
- Non-human furcocercous cercaria
- Parthenita
- Xiphidiocercaria
- Non Human schistosome
